# Intra-voxel incoherent motion MRI of the living human foetus: technique and test–retest repeatability

**DOI:** 10.1186/s41747-017-0031-4

**Published:** 2017-12-22

**Authors:** András Jakab, Ruth Tuura, Raimund Kottke, Christian J Kellenberger, Ianina Scheer

**Affiliations:** 1 Center for MR-Research, University Children’s Hospital, Steinwiesstrasse 75, 8032 Zurich, Switzerland; 2 Computational Imaging Research Lab (CIR), Department of Biomedical Imaging and Image-guided Therapy, Medical University of Vienna, Lazarettgasse 14, 1090 Vienna, Austria; 3 Department of Diagnostic Imaging, University Children’s Hospital, Steinwiesstrasse 75, 8032 Zurich, Switzerland

**Keywords:** Diffusion-weighted imaging (DWI), Foetus, Intra-voxel incoherent motion (IVIM), Magnetic resonance imaging (MRI), Repeatability (reproducibility), Test–retest variability

## Abstract

**Background:**

Our purpose was to test the within-subject (test–retest) reproducibility of the perfusion fraction, diffusion coefficient, and pseudo-diffusion coefficient measurements in various foetus organs and in the placenta based on the intra-voxel incoherent motion (IVIM) principle.

**Methods:**

In utero diffusion-weighted IVIM magnetic resonance imaging (MRI) was performed in 15 pregnant women (pregnancy age 21–36 weeks) on 1.5-T and 3.0-T clinical scanners with b-factors in the range of 0–900 s/mm^2^ in 16 steps. A bi-exponential model was fitted on the volume-averaged diffusion values. Perfusion fraction (f), diffusion coefficient (d), and pseudo-diffusion coefficient (D*) were calculated. Within-subject reproducibility was evaluated as test–retest variability (VAR %) of the IVIM parameters in the foetal frontal cortex, frontal white matter, cerebellum, lungs, kidneys, liver, and in the placenta.

**Results:**

For the foetal lungs, liver and the placenta, test–retest variability was in the range of 14–20% for f, 12–14% for d, and 17–25% for D*. The diffusion coefficients of the investigated brain regions were moderately to highly reproducible (VAR 5–15%). However, f and D* showed inferior reproducibility compared to corresponding measures for the lungs, liver, and placenta. The IVIM parameters of the foetal kidney were revealed to be highly variable across scans.

**Conclusions:**

IVIM MRI potentially provides a novel method for examining microvascular perfusion and diffusion in the developing human foetus. However, reproducibility of perfusion and diffusion parameters depends greatly upon data quality, foetal and maternal movements, and foetal-specific image post-processing.

## Key points

- Foetal IVIM imaging portrays foetal organ and placental microvascular perfusion
- Repeatability of IVIM-derived values are moderate for the placenta, foetal lungs and liver
- Current protocols do not allow repeatable foetal brain and kidney IVIM measurements
- Foetal IVIM imaging requires advanced image post-processing and analysis

## Background

Biological tissues exhibit complex diffusion characteristics due to the presence of multiple micro-scale anatomical compartments and structural barriers. During diffusion-weighted imaging (DWI) obtained with magnetic resonance imaging (MRI) [1], this may result in multiple diffusion coefficients coexisting in the elementary imaging units, so that the most commonly used mono-exponential model of diffusion may not unambiguously represent the underlying physiological phenomena. The intra-voxel incoherent motion (IVIM) concept describes micro-scale translational movements within imaging voxels with a bi-exponential model. While thermally driven Brownian motion results in relatively low apparent diffusion coefficients in human tissues, water protons undergo an order of magnitude larger displacement per unit time as a result of their perfusion-driven flow across the microvascular network. The possible applicability of the IVIM concept in diagnostic imaging was initially suggested in 1989 by Le Bihan et al. [2, 3]. More recently, faster MRI sequences have paved the way for clinical applications of IVIM [4–6].

IVIM relies on the assumption that the fast-moving component arises from blood flowing across the vascular bed in such a way that it mimics a random—incoherent—walk. Studies based on IVIM report that the separation of diffusion and perfusion allows more accurate estimation of tissue diffusivity, quantified as the slow diffusion coefficient or real diffusion and indicated as *d* or slow apparent diffusion coefficient (*ADC*_*slow*_) in organs that are intrinsically highly perfused, such as the liver, kidney, or placenta [7–10]. The diffusion coefficient corresponding to the fast, perfusion-driven component is regarded as the pseudo*-*diffusion coefficient or fast diffusion coefficient: D* or *ADC*_*fast*_, while the perfusion fraction (*f*) describes the fraction of incoherent signal arising from the vascular compartment in each voxel. The parameter *f* is more likely to represent the relative amount of blood flowing through the vascular bed rather than the flow velocity itself [2]. Preliminary studies demonstrated the use of the IVIM imaging in pregnancy [11] by characterising the effect of pathological conditions on the IVIM parameters, such as the alteration of the perfusion fraction associated with intrauterine growth retardation [12–14]. Similarly, arterial spin labelling by means of flow-sensitive alternating inversion recovery has been shown to offer a method for evaluating the transit of blood across the placenta [13].

The successful adaptation of the IVIM technique to the prenatal imaging setting would have important implications for clinical decision-making. For example, the perfusion in the microvascular compartment of the developing foetal lungs, brain, kidneys, or other organs may serve as an indicator of vascular development and organ viability. However, the acquisition of high-quality imaging data with DWI or other echo-planar imaging-based sequences in utero is extremely challenging due to subject motion and the complex anatomical and biochemical environment.

While previous prenatal studies focused on foetal DWI with relatively high b-factors (e.g. 500–800 s/mm^2^) [7, 15–19], we assume that additional lower b-factors may allow the separation of real diffusion and perfusion effects, improving the specificity for pathological changes in microvascular perfusion of the foetal organs in utero. Thus, our purpose was to test the within-subject (test–retest) repeatability of the perfusion fraction, diffusion coefficient, and pseudo-diffusion coefficient in various foetal organs and in the placenta.

## Methods

### Study design

This pilot study was conducted by retrospective enrolment of patients between January 2016 and July 2017. The mothers gave written informed consent for the use of their clinical data for research purposes prior to the examination. The study was approved by the regional ethics committee in Zürich (decision number: 2017-00167).

### Patients

Foetal MRI, including within-session repetition of an IVIM sequence, was performed in 15 pregnancies (maternal age 33.7 ± 5.2 years (mean ± standard deviation [SD], range 24.6–40.8 years). In two cases, a follow-up foetal MRI was performed at two different time points during gestation with 2 and 2.5 weeks between scans and each follow-up scan included with-session repeated IVIM scans. These two measurements of the same cases were treated as independent samples, resulting in 17 IVIM datasets in total for repeatability analysis. The gestational age of the foetuses in the repeatability analysis was 26.3 ± 3.7 weeks (range 21–36 weeks). Foetal MRI was clinically indicated in all cases to rule out or confirm suspected pathologies detected during prenatal screening by ultrasonography. The clinical indication for MRI was isolated mild cerebral ventriculomegaly (*n* = 5), myelomeningocele (*n* = 8), sacrococcygeal teratoma (*n* = 1) and congenital bronchial atresia (*n* = 1).

To illustrate the IVIM technique in clinically relevant pathologies, we included two cases without within-session or across-gestation repeated IVIM data. These two foetuses were diagnosed: one with congenital diaphragmatic hernia (gestational age = 28 weeks) and the other with congenital cystic adenomatous malformation of the lungs (gestational age = 33 weeks).

### MRI protocol

Foetal MRI was performed on two different clinical systems as part of the routine clinical assessment: 12 datasets with a 1.5-T Discovery MR450 unit, six datasets with a 3.0-T Discovery MR750 unit (General Electric Healthcare, Milwaukee, WI, USA). The assignment of the cases to an individual scanner was not controlled in the current study, but based on the availability of free scanner time. IVIM data were collected from January 2016 until March 2017 at the University Children’s Hospital Zürich. Pregnant women were examined in the supine position, feet first, and no contrast agents or sedatives were administered. In order to obtain optimal signal from the foetal head and body within the same session, the coil was readjusted to the position of the foetal structures investigated.

For each foetus, the IVIM imaging sequence was repeated twice with identical settings. The sequence relied on a DWI sequence optimised for foetal imaging, modified to accommodate more b-factors within a clinically feasible imaging time. Slices were positioned in the axial plane relative to the foetal brainstem for brain imaging and in the coronal plane relative to the foetal body for other organs and the placenta.

A dual spin-echo echo-planar sequence was used with echo time/relaxation time of 2200/75 ms, acquisition matrix 80 × 100, voxel size 2 × 2 mm, slice thickness 3 or 4 mm, slice gap 0.5 mm, number of slices 8–14, and 1 excitation. The tetra (tetrahedral) DW orientation scheme was used, which utilises four different combinations of x, y, and z diffusion gradients [20]. b-factor values were increased in 16 steps and one b_0_ image was acquired (b-factors: 0, 10, 20, 30, 40, 60, 80, 100, 150, 200, 300, 400, 500, 600, 700, 800 and 900 s/mm^2^). This scheme resulted in 64 DW images and one b_0_ image for each IVIM image series. The actual imaging time depended on the number of slices, which was adjusted to the size of the foetus and to the focus of the investigation, or specifically whether the brain (8–12 slices) or the whole foetal body and placenta (10–15 slices) were the most important organs for clinical decision-making. Imaging time per IVIM acquisition ranged from 1 min 40 s to 3 min 20 s.

### IVIM post-processing

Post-processing was carried out using an in-house developed script written in BASH language for Linux. It utilised image processing algorithms from the Functional Magnetic Resonance Imaging of the Brain (FMRIB) Software Library (FSL) [21], C3D [22], and NIFTIREG [23] software packages for image registration and re-sampling. The image analysis script is available as online supplement material to this manuscript.

**Fig. 1:**
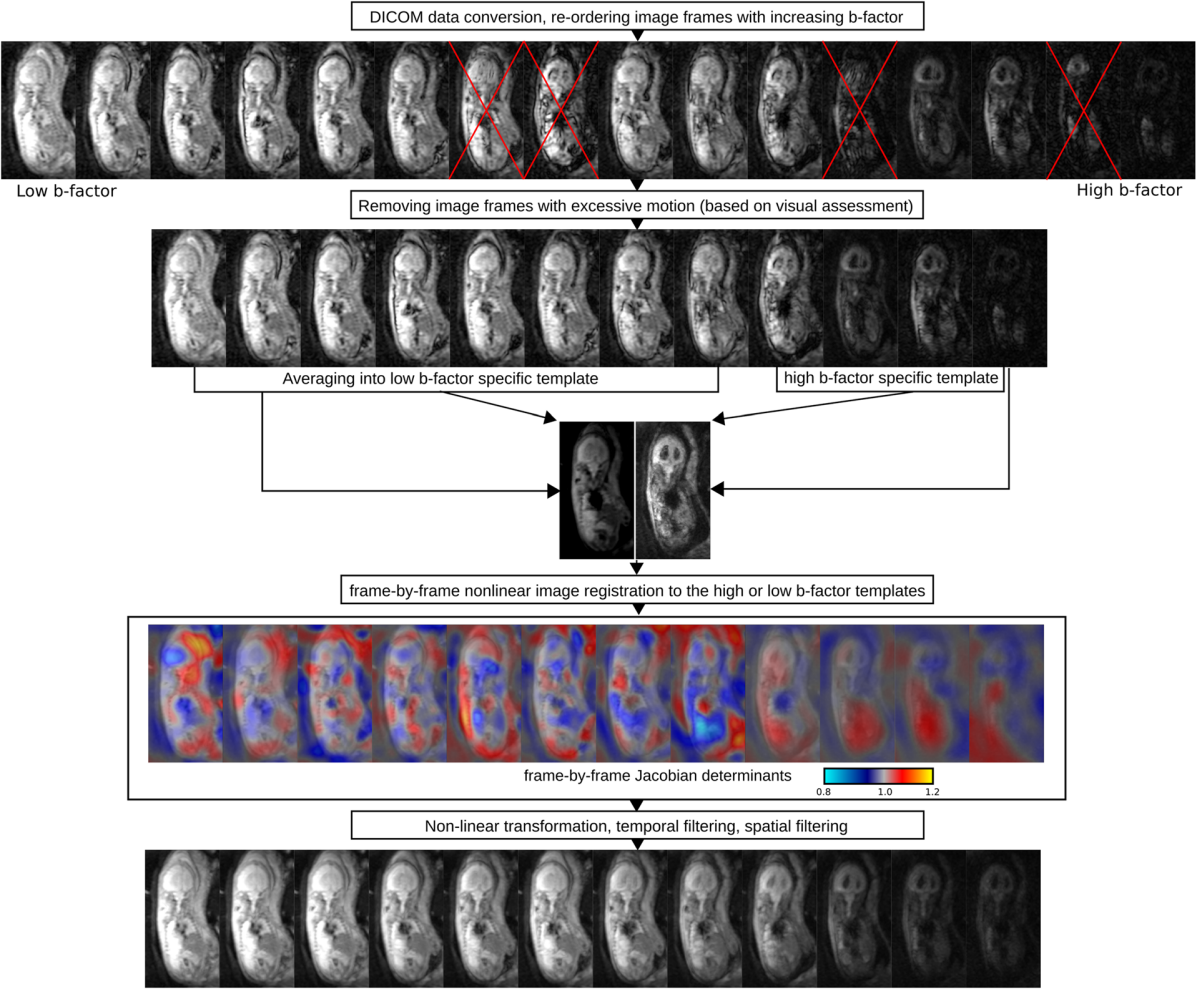
Post-processing steps to correct fetal in utero IVIM datasets for subject motion

First, the raw IVIM data were viewed using the *fslview* command of the FSL software and the image frames with the most excessive subject motion were marked and removed from the analysis. This step was followed by a non-linear, free-form deformation-based registration of image frames with the *reg*_*f3d* command in the NIFTIREG tool, the registration steps of which are illustrated in Fig. 1. This image registration step used a fine deformation grid with a grid spacing of 6 × 6 × 6 mm for the low b-factor image frames and 12 × 12 × 12 mm grid for the high b-factor frames.

### Volume of interest definition

**Fig. 2:**
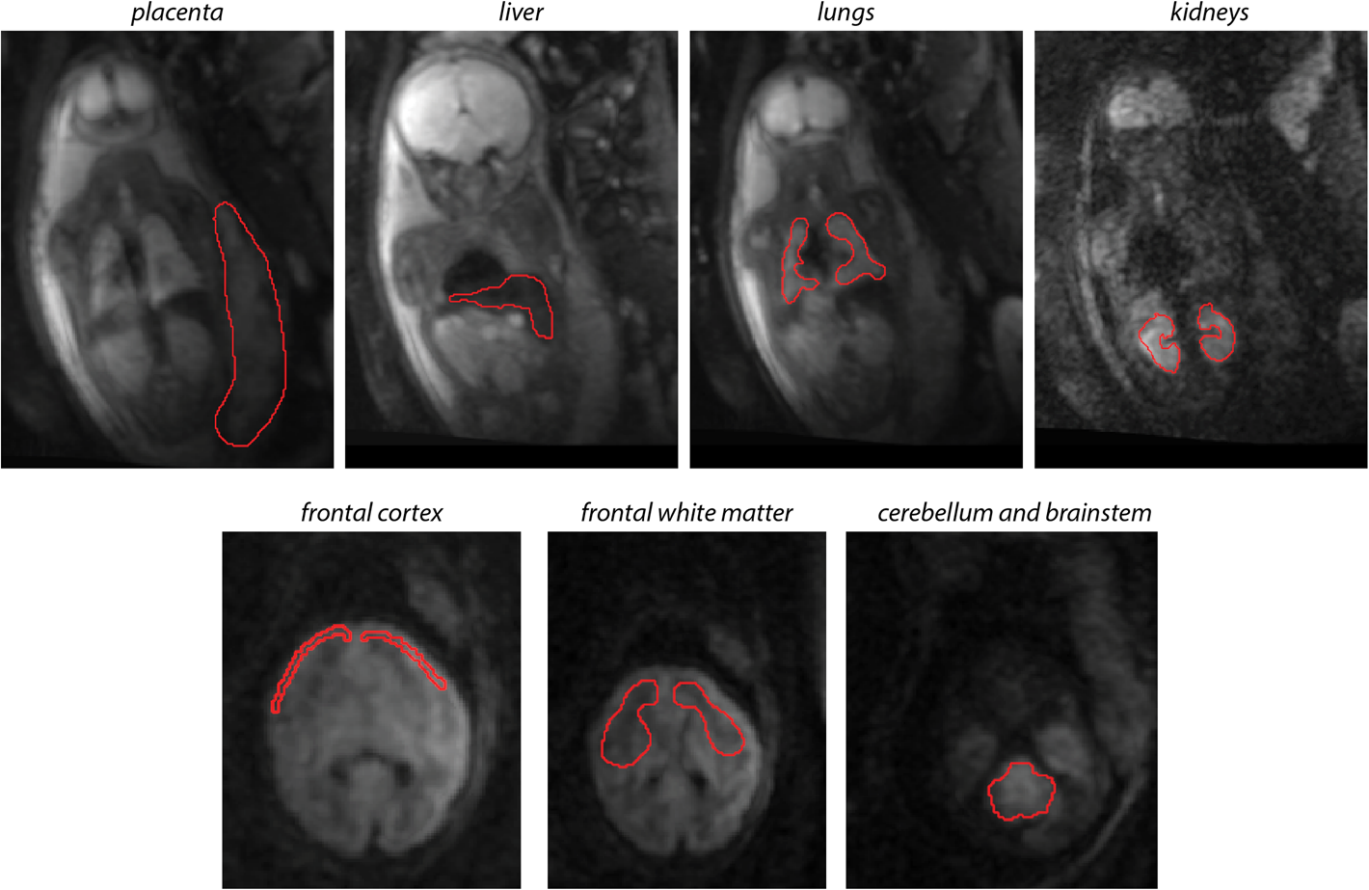
VOI delineation of various foetal organs and the placenta. VOIs have been manually delineated to test the within-subject repeatability of the parameters that are calculated from IVIM data. *Red overlay*: manual outlines of the organs. Background image: coronal or axial DW images

After processing IVIM data, averaged images for low b-factors (b < 250 s/mm^2^) and high b-factors (500–900 s/mm^2^) were generated. Using the manual segmentation tool in the medical imaging interaction toolkit (MITK) [24], volumes of interest (VOIs) were placed over the central part of the placenta, on the foetal liver, lung parenchyma excluding the hili, kidneys bilaterally, cerebellum and brainstem, frontal or frontoparietal cortical mantle, and white matter of the frontal and parietal lobes (Fig. 2). All VOIs were drawn manually by one observer with four years of experience in foetal MRI. Three-dimensional interpolation in the MITK software was then used to smooth the borders of the delineated organ labels. For the kidneys and the placenta, better visual discrimination from surrounding tissues was achieved by delineating the VOIs by viewing the diffusion images with higher b-factors, while for the other structures we used the diffusion images averaged over lower b-factors.

### IVIM model fitting

The IVIM parameters *f*, *d*, and D* were estimated based on the VOI-averaged signal intensity values to achieve a better signal-to-noise ratio (SNR). The analysis of diffusion and perfusion parameters with the IVIM model assumed two compartments without interactions [5]. A bi-exponential model (Eq. 1) was fitted in two steps on the averaged signal intensity over the VOIs:

1 $$ \frac{S}{S_0}=f\ast {e}^{-b{D}^{\ast }}+\left(1-f\right)\ast {e}^{- bd} $$

where *S* is the measured signal intensity, *S*_0_ is the signal intensity without diffusion-weighting, *d* is the diffusion coefficient, D* is the pseudo diffusion coefficient, *f* is the perfusion fraction, and *b* is the b-factor.

First, the measurements were fitted for b-values higher than 250 s/mm^2^ to estimate the parameter *d* using a mono-exponential term. Then, *f* and D* were estimated keeping *d* fixed at the previously fitted value. The IVIM model fitting was carried out with the MITK diffusion toolkit.

### Repeatability analysis

Repeatability of *f*, *d*, and D* over the repeated scans was measured as the test–retest variability:

2 $$ VAR\%={100}^{\ast}\frac{1}{N}\sum \limits_{i=1}^N\frac{\left| TES{T}_i- RETES{T}_i\right|}{\left( TES{T}_i+ RETES{T}_i\right)/2} $$

where *N* is the number of individuals and *TEST*_*i*_ and *RETEST*_*i*_ are the duplicate measurements for subject *i*.

Next, we tested whether the variability of the IVIM parameters is affected by possible confounds. Multiple, univariate analysis of variance (ANOVA) was carried out with the ‘General linear model’ module in SPSS v22.0 for Windows (Mathworks inc., Nattick, MA, USA). In this analysis, the test–retest difference (that is: $\frac{\left| TES{T}_i- RETES{T}_i\right|}{\left( TES{T}_i+ RETES{T}_i\right)/2}$) of *f*, *d*, and D* of each organ served as the dependent variable. We evaluated the effect of gestational age, maternal age, scanner field strength, and number of removed image frames on the test–retest difference of each IVIM parameter of the investigated organs. To reveal interactions between the IVIM parameters and the assumed confounds, we report results of the ANOVA tests. Values of *p* < 0.050 were considered significant.

## Results

### IVIM characteristics of fetal tissues and placenta

**Fig. 3:**
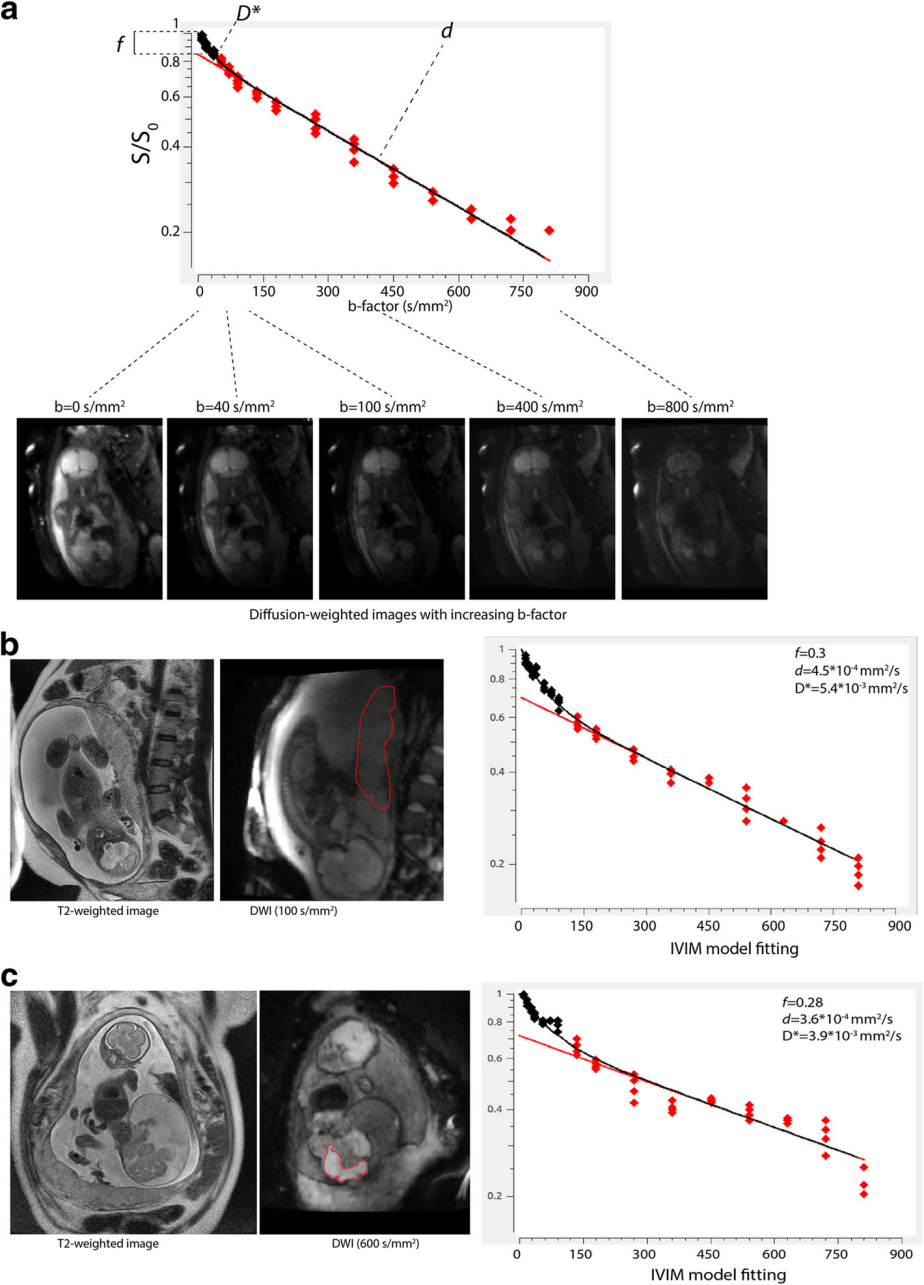
IVIM imaging in utero. **a** Bi-exponential model fitting on the DWI measurements that have been acquired with increasing b-factor. **b** IVIM imaging of the placenta. *Left*: coronal T2-weighted image; *middle*: DW image (placenta delineated); *right*: IVIM signal and estimates of the diffusion coefficient *d*, pseudo-diffusion coefficient D* and the microvascular perfusion fraction *f*, based on VOI-averaged values. **c** IVIM image of a sacrococcygeal teratoma

The typical appearance of the DW foetal images using an IVIM sequence in well-perfused organs is shown in Fig. 3a: increasing the b-value, the signal intensity decreases exponentially, especially with b > 250 s/mm^2^. In the lower b-factor range (<250 s/mm^2^), the faster diffusion determines a higher S/S_0_ ratio than that would be expected by extrapolating the mono-exponential fitting (black dots and black regression line). The IVIM images in the low b-factor range show predominantly T2 characteristics and most foetal organs were easy to delineate. With increasing b-factor, only the brain, kidneys, placenta, and the muscular layer of the uterus remained distinguishable from the background noise.

**Fig. 4:**
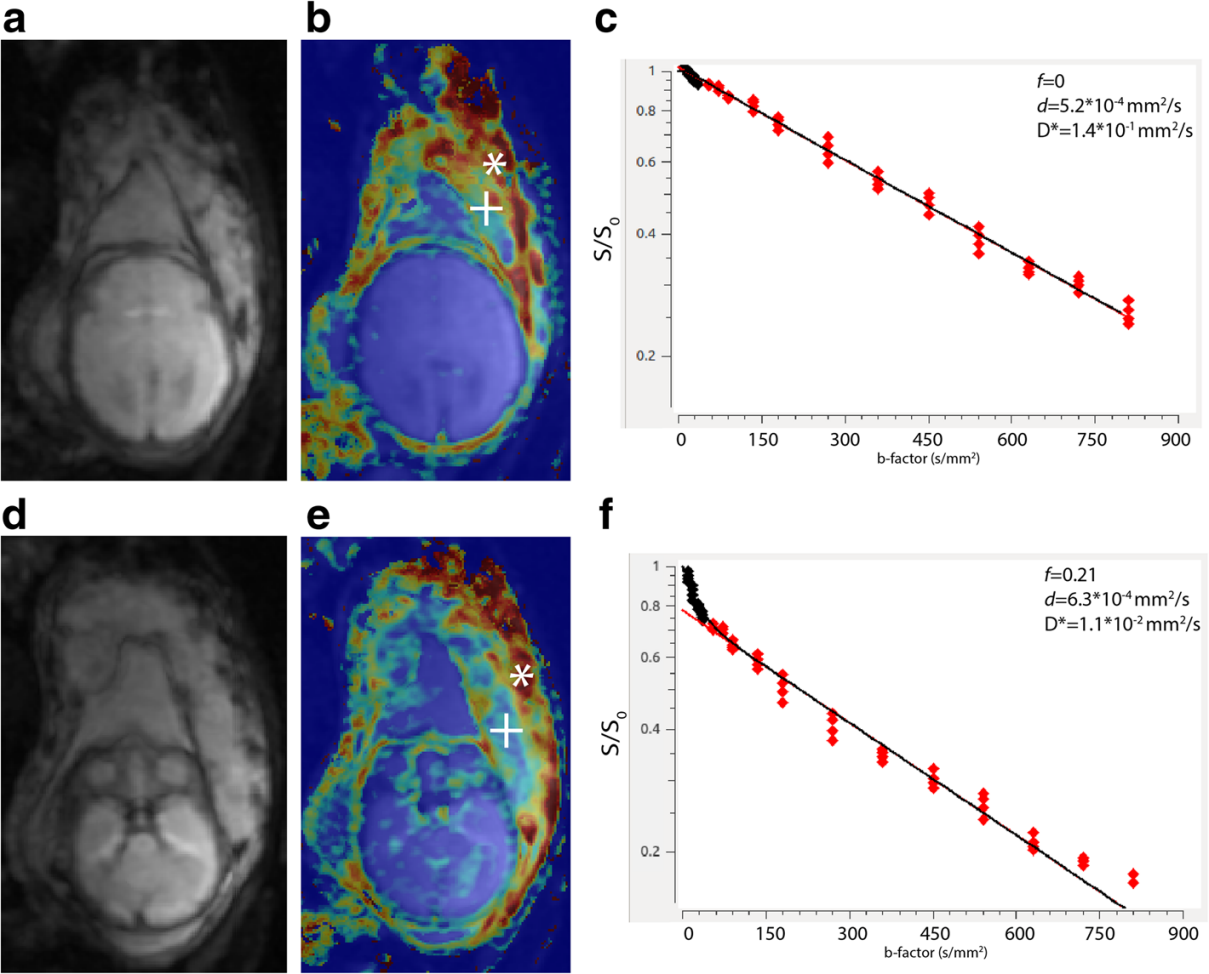
Foetal brain. **a** DW axial images showing the foetal brain at the level of the third ventricle. **b** Perfusion fraction map at the same level. **c** IVIM model fitting curve of the foetal brain based on a frontal white matter VOI. **d** DW axial images showing the foetal brain and the central part of the placenta. **e** Perfusion fraction map at the same level. **f** IVIM curve of the central placenta. *Cross*: central part of the placenta, *asterisk*: basal plate of the placenta

We found a high microvascular perfusion fraction in the foetal liver (*f* = 0.346 ± 0.101, mean ± SD) and lungs (*f* = 0.33 ± 0.112). The liver appeared as a homogeneously and highly perfused organ on the IVIM parametric maps. The central (hilar) parts of the lungs displayed a higher *f* than their periphery, while *f* was not as prominently high as in the adjacent heart and great vessels, which typically had *f* values over 0.5. The central part of the placenta was moderately and homogeneously perfused (population average *f* = 0.28 ± 0.105), with a tendency towards a higher perfusion near the basal layer (Fig. 4b and e, asterisk). The kidneys were also moderately perfused (*f* = 0.153 ± 0.09). We found high heterogeneity and putative partial volume artifacts caused by the movement of these organs relative to the imaging plane due to maternal breathing, foetal breathing, and foetal trunk movements.

**Fig. 5:**
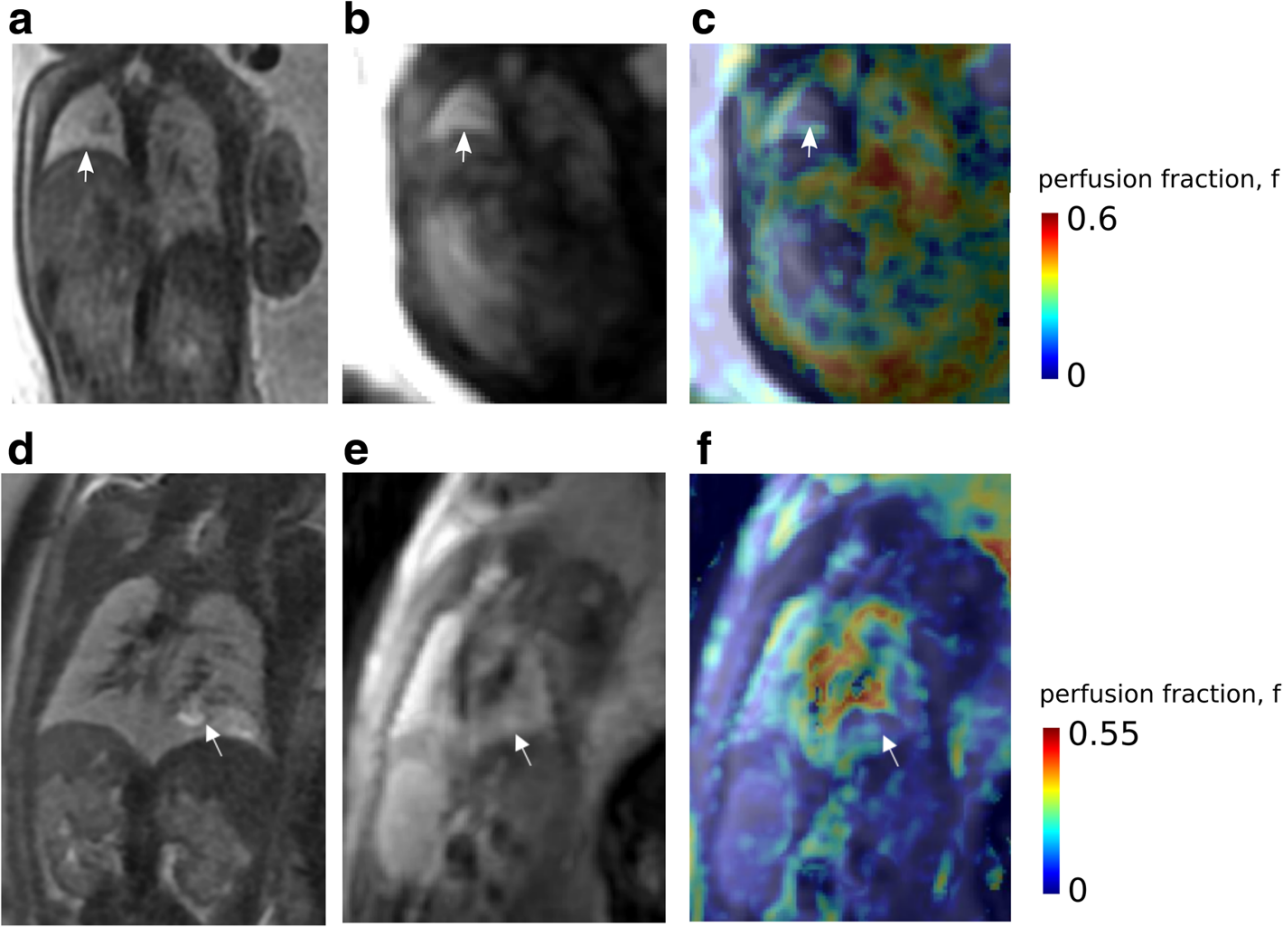
**a**–**c** Congenital diaphragmatic hernia: hypoplastic left lung (*arrow*); normal right lung. **a** T2-weighted coronal image. **b** DWI, b-factor = 0 image. **c** IVIM perfusion fraction map showing decreased microvascular perfusion fraction in the hypoplastic left lung. **d**–**f** Congenital cystic adenomatoid formation of the right lung. **d** Coronal T2-weighted image showing a hyperintense abnormality at the inferior lobe of the right lung. **e** DWI, b-factor = 0 image. **f** IVIM perfusion fraction map showing a demarcated zone of decreased microvascular perfusion fraction in the affected region of the inferior lobe (*arrow*)

We investigated two lung pathologies: a congenital diaphragmatic herniation and a cystic adenomatoid malformation of the lung (Fig. 5). The hypoplastic lung in congenital diaphragmatic herniation demonstrated a global reduction of the microvascular perfusion fraction (Fig. 5c). Despite the low SNR, the microvascular perfusion map in the foetal lungs in the cystic adenomatoid malformation *f* was decreased locally over a demarcated spot in the inferior lobe of the lung (Fig. 5f), this spot being co-localised with the hyperintensity on the T2-weighted image (Fig. 5d).

All the three brain regions demonstrated a low microvascular perfusion (frontal white matter, *f* = 0.087 ± 0.106; frontal cortex, *f* = 0.142 ± 0.133; cerebellum, *f* = 0.135 ± 0.122). Interestingly, in many cases *f* and D* in the foetal brain were estimated to be 0, which is most likely an artefact due to the insufficient data quality for estimating low perfusion values, or D* was estimated to be smaller than *d*. Compared to the neighbouring central placenta or basal plate (Fig. 4f), the foetal brain, especially the white matter, appeared to be almost non-perfused (Fig. 4c). We measured higher *f* values in the foetal brain frontal cortex; however, this is likely to arise from partial volume effects with the adjacent cerebrospinal fluid spaces, which inherently displayed a sharp signal decay in DWI experiments due to the bulk movement of proton spins.

### Within-subject (test–retest) repeatability

**Fig. 6:**
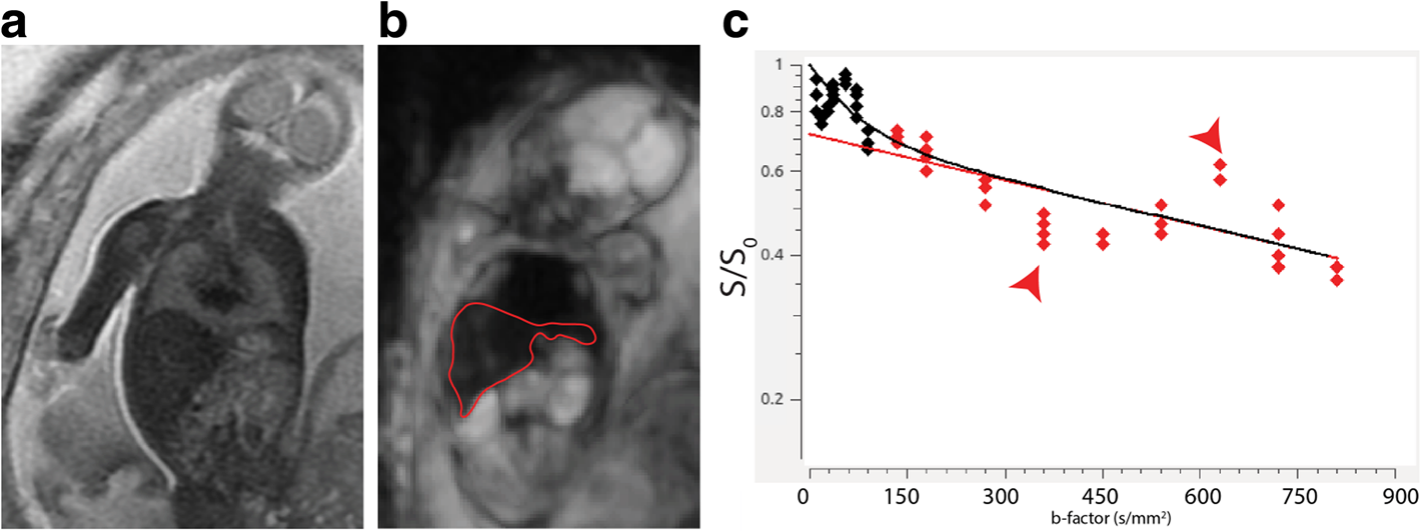
IVIM imaging of a foetus that has moved considerably during the acquisition. **a** T2-weighted image. **b** DW image. **c** Bi-exponential fitting based on the IVIM measurements. Sudden changes in position and foetal breathing movements cause particularly large displacements of foetal abdominal organs, such as the liver (*red outline*, *middle image*), and increase or decrease the measured signal intensity (*red arrows*)

Images acquired with this IVIM protocol were prone to three main sources of motion and consequent artifacts (maternal breathing, foetal body movements, and physiological movements of foetal internal organs), which can be identified by looking at the raw DW images or observing the outlier points of the bi-exponential curve fitting (Fig. 6c).

The effect of large movements of the foetal body was partially mitigated by removing 6.1 ± 7.4 image frames with excessive motion before the analysis. Of the IVIM parameters, the diffusion coefficient showed the highest repeatability for all the investigated foetal structures and the placenta in terms of VAR% (frontal cortex 4.8%, placenta 12.2%), as estimated by the mono-exponential decay of signal intensities corresponding to image frames with a b-factor > 250 s/mm^2^. In contrast, *f* and D* values were twice as variable across repeated scans as was *d*. Only three organs, the placenta, foetal liver and lungs were found to show moderately repeatable perfusion fractions and pseudo-diffusion coefficients (Table 1), while *f* and D* of brain areas and kidneys showed a poor repeatability, with a test–retest variability over 25%.

**Table 1:** Foetal IVIM parameters and their repeatability across the study population

Structure and number (n) of individuals where the structure was repeatedly imaged	IVIM measurements			Within-subject variability (VAR%)			Subjects where bi-exponential fitting was insufficient, *f* and D* = 0 (n (%))
	Perfusion fraction *f*	Diffusion coefficient *d* (mm^2^/s)	Pseudo-diffusion coefficient D* (mm^2^/s)	Perfusion fraction *f* (%)	Diffusion coefficient *d* (%)	Pseudo- diffusion coefficient D* (%)	
Brain, frontal cortex (*n* = 11)	0.142 ± 0.133 (0–0.54)^a^	4.7 × 10^–4^ ± 9.9 × 10^–5^(2.9 × 10^–4^–7.7 × 10^–4^)	9.5 × 10^–3^ ± 2.9 × 10^–2^(1 × 10^–3^–1.5 × 10^–1^)	23.5	4.8	19.9	2 (18.2)
Brain, frontal white matter (*n* = 8)	0.087 ± 0.106 (0–0.377)^a^	5.2 × 10^–4^ ± 1.3 × 10^–4^(2.9 × 10^–4^–7.8 × 10^–4^)	7.3 × 10^–3^ ± 2.8 × 10^–2^(1 × 10^–3^–1.5 × 10^–1^)	33.6	12.8	27.9	5 (62.5)
Cerebellum (*n* = 9)	0.135 ± 0.122 (0–0.419)^a^	4.6 × 10^–4^ ± 1.1 × 10^–4^(2.3 × 10^–4^–6.9 × 10^–4^)	9.4 × 10^–3^ ± 3.0 × 10^–2^(1 × 10^–3^–1.5 × 10^–1^)	33.5	15.2	28.2	3 (33.3)
Placenta (*n* = 16)	0.28 ± 0.105 (0–0.479)^a^	5.4 × 10^–4^ ± 8.2 × 10^–5^(2.9 × 10^–4^–6.8 × 10^–4^)	4.7 × 10^–3^ ± 3.8 × 10^–3^(2.5 × 10^–3^–2.3 × 10^–2^)	16.9	12.2	17	1 (6.3)
Liver (*n* = 8)	0.346 ± 0.101 (0.149–0.479)	3.7 × 10^–4^ ± 8.7 × 10^–5^(2.3 × 10^–4^–5.9 × 10^–4^)	2.6 × 10^–3^ ± 2.4 × 10^–3^(0–9.9 × 10^–3^)	14.4	13.8	16.8	0 (0)
Lung (*n* = 9)	0.33 ± 0.112 (0.108–0.548)	5.5 × 10^–4^ ± 9.2 × 10^–5^(4 × 10^–4^–8.1 × 10^–4^)	2.5 × 10^–3^ ± 1.3 × 10^–3^(1 × 10^–3^–5.5 × 10^–1^)	20.4	14.1	25.3	0 (0)
Kidneys (*n* = 5)	0.153 ± 0.09 (0–0.307)^a^	4.2 × 10^–4^ ± 8.6 × 10^–5^(3.1 × 10^–4^–6.4 × 10^–4^)	1.7 × 10^–2^ ± 4.2 × 10^–2^(1 × 10^–3^–1.5 × 10^–1^)	36.2	17	30.1	2 (40)

Data are given as mean ± SD and range, while repeatability is expressed as test–retest variability (VAR%, see Eq. 2) of repeated measurements

^a^Cases where the number for assessing the variability of *f* was further reduced due to the insufficient fitting of the bi-exponential curve (in such cases, *f* and D* were equal to zero); the number of missing data entries is given in the last column of the table

### Factors influencing the repeatability of IVIM parameters

In our experiments, the number of frames removed was indicative of the subject motion; a positive correlation was found between the within-subject variability of two IVIM parameters and the number of frames removed. The number of frames removed also influenced the repeatability of the diffusion coefficient of the frontal cortex (ANOVA, *F* = 6.28, *p* = 0.046, *β* = 0.0032) and that of the perfusion fraction of the cerebellum (ANOVA, *F* = 25.68, *p* = 0.007, *β* = 0.026). Fetuses at later ages of gestation showed a higher within-subject variability of the perfusion fraction of the cerebellum (ANOVA, *F* = 14.625, *p* = 0.019, *β* = 0.0042). Scanner type (1.5-T or 3.0-T field strength) and maternal age were not found to correlate with any of the repeatability measurements.

## Discussion

Within-subject, repeated in utero IVIM from 15 pregnancies demonstrated that *f*, *d*, and D* can be reliably measured in foetal lungs, liver, and placenta. For these organs, within-subject variability during test–retest imaging was in the range of 14.4–20.4% for *f*, 12.2–14.1% for *d*, and 16.8–25.3% for D*. The diffusion coefficients of the investigated brain regions were moderately to highly repeatable (variability of 4.8–15.2%). However, *f* and D* showed inferior repeatability compared with corresponding measures derived for lungs, liver, and placenta. The IVIM-based parameters of the foetal kidney were revealed to be highly variable across scans.

### Quality of foetal IVIM data: repeatability and confounds

The adaptation of diffusion MRI techniques to the foetal age faces numerous challenges [25]. The IVIM approach is based on an echo-planar sequence, more susceptible to image artifacts than standard anatomical MRI [26].

Our study adds more knowledge to the body of previous reports evaluating the reproducibility of the IVIM-derived parameters [27] and extends them by providing initial results about repeatability in utero. An important part of our analysis tested whether acquisition-related or subject-related confounding factors cause significant variability in tissue diffusivity and perfusion. The slow diffusion coefficient *d*—the parameter most commonly referred to as ADC in clinical studies—was the most repeatable (VAR%, placenta 4.8, liver 13.8%). This finding is in good agreement with previous abdominal IVIM studies reporting that *d* is twice as reproducible as the microvascular perfusion fraction *f* or D* [8]. This is partially due to the fact that the mono-exponential component is estimated by us using numerous measurement points with higher, well separated diffusion-weightings (we used ten measurement points for high b-values, Fig. 3a). Images acquired with higher b-values are less prone to the effects of T2 relaxation [28].

The pseudo-diffusion coefficient D* and microvascular perfusion fraction *f* were twice as variable as *d*, highlighting the uncertainty in estimating the faster diffusion component, as previously reported [29]. The lower SNR in estimating the fast diffusion component reduces the diagnostic value of such parameters and a further confounding factor arises from the impossibility to reach the desired low diffusion-weighting because of limitations of the scanner hardware [26]. Regarding repeatability, considerable differences were found between foetal organs, with the brain (frontal cortex, white matter, and cerebellum) and the kidney having insufficient repeatability for further analysis. The foetal kidneys were exceedingly prone to motion-related artifacts and their small size putatively increased partial volume errors.

The brain in the developing foetus appeared to have very low D* and *f* values, which can reflect low microvascular perfusion. In the adult brain, the capillary blood volume fraction is known to be very low (2–4%) compared to that in other organs [5, 6], determining the need for a very good SNR to reproducibly quantify D* and *f*. Furthermore, the immaturity of cerebral capillary vasculature network in the foetus may contribute to the observed low D* and *f* values and their low reproducibility. In mid-gestation, the developing cortex and subcortex show lower vessel density and vasculature is more dominated by penetrating arteries running orthogonal to the pial surface rather than long-range cortico-cortical vessels [30]. This may breach one of the important assumptions of IVIM imaging, namely the presence of randomly oriented vascular segments within the imaged voxels, resulting in a low IVIM signal in the foetal brain. However, the most likely explanation for the brain low (or zero) IVIM values lies in the poor data quality of these measurements. For the brain, placenta, and kidney, *f* was estimated to be zero due to the insufficient fitting of the bi-exponential function from 6.3% of the cases for placenta to 62.5% of the cases for frontal white matter.

We identified a number of additional limiting factors affecting IVIM data quality. The availability of data for test–retest repeatability was restricted by the limited visibility of some of the organs due to the selective placement of the imaging field of view. Liver and lung microvascular perfusion fractions were estimated to be larger on the 3.0-T magnet compared to the 1.5-T magnet, as previously reported [4, 31, 32]. This might be the consequence of the dependence of microvascular perfusion fraction on the echo time [4], since longer echo times cause greater signal decay at low b-values. MRI at 3.0 T is associated with larger magnetic field inhomogeneity and more susceptibility artifacts, which are exaggerated by the complex chemical environment of the amniotic fluid, the maternal organs and skeletal structures [33]. Multicentre studies [34, 35] have revealed a larger inter-scanner variability than intra-scanner variability for diffusion measures, which calls for a careful interpretation of studies conducted on different scanners. To overcome this limitation, VOI-based or organ-based estimation of IVIM parameters should be used instead of voxel level curve fitting. It was also shown that the quality of IVIM parameters is greatly observer-dependent and additionally depends on sequence parameters and scanner field strength [36] as well as on the mathematical model estimating the parameters [37, 38].

In the in utero setting, the usability is further limited by considerable data dropout. The need to visually control each and every image frame for motion artifacts before image post-processing limits the applicability of the method for diagnostic purposes. This step during the processing work-flow would optimally be replaced with the automatic evaluation of frame-to-frame motion based on similarity metrics [39], but such metrics are challenging to implement in practice due to the gradually changing image contrast with increasing b-values during the acquisition scheme.

In conclusion, IVIM potentially provides a novel method for examining microvascular perfusion and diffusion in the developing human foetus. However, repeatability of perfusion and diffusion parameters depends greatly upon data quality, foetal and maternal movements, and foetal-specific image post-processing.

## Abbreviations

ADC: Apparent diffusion coefficient
ANOVA: Analysis of variance
DWI: Diffusion-weighted imaging
FSL: FMRIB software library
IVIM: Intra-voxel incoherent motion
MITK: Medical imaging interaction toolkit
MRI: Magnetic resonance imaging
SD: Standard deviation
SNR: signal-to-noise-ratio
VAR: Variability
VOI: Volume of interest
